# Nasopharyngeal Chondrolipoma

**DOI:** 10.1155/2010/838046

**Published:** 2010-06-08

**Authors:** A. J. Kinshuck, S. Agrawal, V. M. Patel, P. W. Bishop, P. H. Jones

**Affiliations:** ^1^Department of Otolaryngology and Head & Neck Surgery, University Hospital Aintree, Longmoor Lane, Liverpool L9 7AL, UK; ^2^Department of Otolaryngology, Wythenshawe Hospital, University Hospital of South Manchester NHS Foundation Trust, Manchester M23 9LT, UK; ^3^Department of Pathology, Wythenshawe Hospital, University Hospital of South Manchester NHS Foundation Trust, Manchester M23 9LT, UK

## Abstract

In this case report, we describe the presentation and treatment of a patient with nasopharyngeal chondrolipoma. Lipomas are common soft tissue tumours, although their incidence in the nasopharynx is very low. A rarer variant of lipoma, chondrolipomas are benign mesenchymal tumours. They are formed by the proliferation of mature adipocytes and contain different amounts of mature cartilaginous tissue; Weiss “Enzinger and Weiss's soft tissue tumours”, 4th ed: Mosby, St Louis; 2001 This represents the second reported case of a nasopharyngeal chondrolipoma. An endonasal approach to excision has not been previously described.

## 1. Introduction

There are few reported cases of lipomas arising in the nasopharynx and only one reported case, of nasopharyngeal chondrolipoma [1]. In the present case we describe a nasopharyngeal chondrolipoma in a 70-year-old male.A chondrolipoma is a lipoma with focal cartilaginous metaplasia. Lipomas are common benign tumours of mesenchymal origin. Lipomas consist of adipocytes arranged in lobules separated by septa of fibrous connective tissue. They may be associated with other mesenchymal elements. Histopathological variants include fibrolipomas, angiolipomas, myolipomas, osteolipomas and chondrolipomas [2, 3].

In this case, it was possible to excise the chondrolipoma via an endonasal endoscopic approach. 

## 2. Clinical Report

A 70-year-old male was referred for ENT assessment with a twelve month history of increasing nasal congestion and globus sensation. There was no history of weight loss, epistaxis or loss of smell. He denied any otological symptoms. 

**Figure 1:**
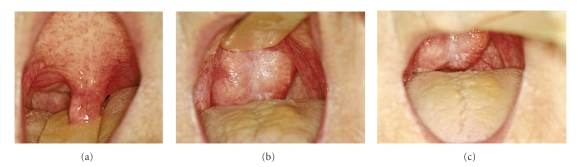
Large nasopharyngeal mass visualised on retraction of soft palate.

Nasoendoscopy demonstrated a mass in the postnasal space. It was not possible to pass the scope beyond the mass due to its size. Oropharyngeal examination revealed a large smooth mass extending beyond the soft palate. It was clearly visible with the soft palate retracted (Figure 1). 

A magnetic resonance scan of the head and neck confirmed the presence of a mass in the postnasal space arising from the right eustachian tube cushion. The scan signal intensities were consistent with a lipoma. 

The patient proceeded to examination under anaesthetic of the nasopharynx. Following nasal decongestion nasoendoscopy demonstrated the mass attached to the post nasal space by a narrow pedicle. Using a transnasal endoscopic approach the pedicle was dissected and divided, allowing the mass to be mobilized and removed via the mouth. It was submitted for histological examination. There was minimal bleeding and nasal packing was not required.

### 2.1. Histology

**Figure 2:**
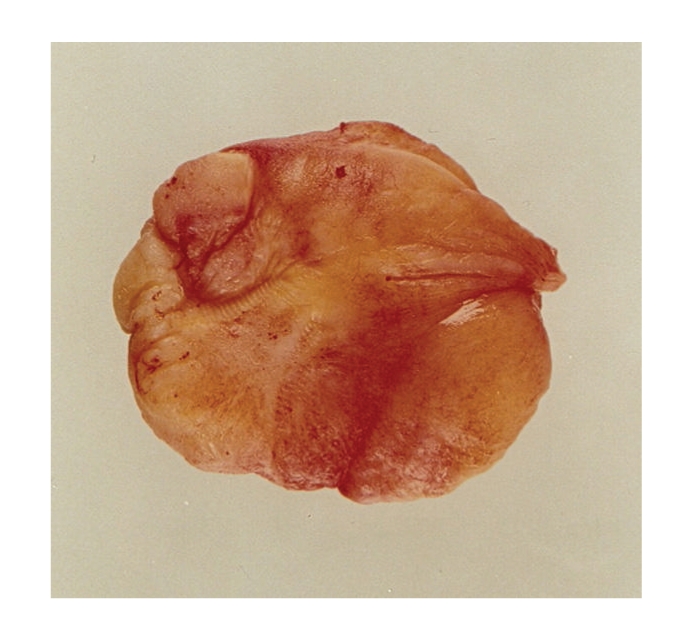
Pathology specimen.

The mass had a lobulated cream appearance with a smooth surface measuring 5 × 4 × 2 cm. The cut surface was cream coloured, gelatinous with a yellow outer rim (Figure 2). 

**Figure 3:**
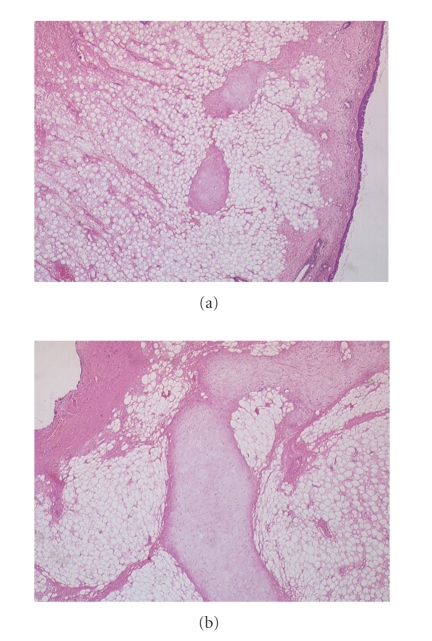
The images above are both at × 20 magnification. The view in Figure 3(a) shows a predominance of mature adipose tissue with fine bands of fibrosis and round islands of cartilage. The surface ciliated columnar epithelium is to be seen on the right of this image along with an island of cartilage. The chondrocytes lack cytological atypia or mitotic activity. Figure 3(b) shows an island of cartilage at higher power.

Microscopically, the mass was covered by a ciliated columnar epithelium showing focal squamous metaplasia. The stroma was composed of fibrous tissue exhibiting myxoid changes and mixed with mature fatty tissue and thin and thick walled blood vessels, some containing thrombi. Islands of cartilaginous tissue were present There were scattered mast cells and perivascular cuffing by lymphocytes and plasma cells. The appearances were concluded to be consistent with a chondrolipoma (Figure 3).

## 3. Discussion

There are a variety of clinical manifestations associated with a nasopharyngeal mass. In this case, the patient presented with nasal obstruction and a globus sensation. Other symptoms may include: anosmia, halitosis, rhinorrhoea, post nasal drip, nasal obstruction, epistaxis, snoring, obstructive sleep apnoea, dysphagia, cervical lymph node enlargement, palatal mass, aural fullness, otalgia, hearing loss, and voice change [4]. 

Lipomas are one of the most common mesenchymal tumours and 13 percent of lipomas occur in the head and neck region [5]. The nasopharynx is a rare site; there have been only five reported cases of lipomas within the nasopharynx described in the literature [6–10]. 

Lipomas are benign tumours consisting of mature fat cells and are single or lobulated. They may be soft, firm, or cystic and may arise in any location where fat is normally present. The growth of lipomas is due to proliferation of cells similar to fibroblasts. Lipomas may contain a variety of mesenchymal elements. These may include fibrous connective tissue (fibrolipoma), myxomatous (myxolipoma), capillary angiomatous tissue (angiolipoma), and mucoid substances (myxoid lipoma) [2, 3].

There has been only one reported case of nasopharyngeal chondrolipoma [1]. Histologically, they are characterized by the proliferation of mature adipocytes associated with the deposition of mature cartilaginous tissue. They appear to occur in large, long-standing lipomas.

The described nasopharyngeal lipomas arise from adipose tissue in the submucosa and are attached by a sessile stalk or pedicle. In the present case, the pedicle was divided under nasoendoscopic guidance.

The investigation of a patient with these symptoms would include a full ENT history and examination including nasoendoscopy. A CT scan may provide a definitive diagnosis by calculating the density of the suspected mass. The CT attenuation number is low due to fat being of lower density than water. MRI scans may also accurately diagnose lipomas preoperatively because of lipomas typical signal intensity patterns [5].

Treatment of nasopharyngeal lipomas is surgical with curative intent. Chondrolipomas are large and well demarcated facilitating their excision. 

## 4. Conclusion

We report a case of a nasopharyngeal chondrolipoma treated by nasoendoscopic dissection and removal. Although rare, it should be considered in patients presenting with symptoms of a nasopharyngeal mass. Cartilaginous metaplasia giving rise to chondrolipomas is found in large, long-standing lipomas. The treatment of these tumours is by surgical excision, following which there have been no reported cases of recurrence.

## References

[1] Hong KH, Seo SY, Lee DG (1998). Chondrolipoma of the nasopharynx. *Journal of Laryngology and Otology*.

[2] Weiss SGJ (2001). *Enzinger and Weiss’s Soft Tissue Tumours*.

[3] Underwood J (2000). *General and Systematic Pathology*.

[4] Piccin O, Sorrenti G (2007). Adult obstructive sleep apnea related to nasopharyngeal obstruction: a case of retropharyngeal lipoma and pathogenetic considerations. *Sleep and Breathing*.

[5] El-Monem MHA, Gaafar AH, Magdy EA (2006). Lipomas of the head and neck: presentation variability and diagnostic work-up. *Journal of Laryngology and Otology*.

[6] Puri ND, Vaid A, Sawhney KL (1979). Fibrolipoma of the nasopharynx. *Journal of the Indian Medical Association*.

[7] Oddie JW, Applebaum EL (1982). Lipoma of the nasopharynx. *Archives of Otolaryngology*.

[8] Grybauskas VT, Shugar MA (1983). Nasopharyngeal lipoma. *Laryngoscope*.

[9] Fagan JJ, Learmonth GM, Garb M, Bowen RM (1996). Nasopharyngeal lipoma—a rare clinico-pathological entity. *Journal of Laryngology and Otology*.

[10] Kalan A, Ahmed-Shuaib A, Tariq M (2000). Lipoma in fossa of Rosenmuller. *Journal of Laryngology and Otology*.

